# Nurses’ Experiences of Managing and Management in a Critical Care Unit

**DOI:** 10.1177/2333393614532617

**Published:** 2014-06-03

**Authors:** K. Robyn Ogle, Nel Glass

**Affiliations:** 1 Australian Catholic University, Melbourne, Victoria, Australia

**Keywords:** critical methods, discourse analysis, ethnography, feminism, health care administration, intensive care unit (ICU), nursing

## Abstract

In this article, we describe the major findings of an ethnographic study undertaken to investigate nurses’ experiences of managing nurses and being managed by nurses in an Australian critical care unit. Our purpose was to valorize and make space for nurses to speak of their experiences and investigate the cultural practices and knowledges that comprised nursing management discourses. Subjugated practices, knowledges, and discourses were identified, revealing how nurses were inscribed by, or resisted, the discourses, including their multiple mobile subject positions. Informed by critical, feminist, and postmodern perspectives, nine mobile subject positions were identified. Direct participant observation, participant interviews, and reflective field notes were analyzed for dominant and subjugated discourses. The major finding described is the subject position of “junior novice.” Nurses informed by dominant patriarchal and organizational discourses participated in constructing and reinscribing their own submissive identity reflected in interprofessional relations that lacked individual valuing and undermined their self-esteem.

There is a worldwide shortage of nurses that is endemic, and significant workforce reform is required (Health Workforce Australia [HWA], 2012). Although nurses make up the largest health care workforce (HWA, 2012), and provide most of the patient care as a gendered segregated occupation (Husso & Hirvonen, 2012), research on nursing culture remains limited (Scott-Findlay & Estabrooks, 2006; Struebert, 2011). The focus has been workforce modeling (HWA, 2012), stress (McGibbon, Peter, & Gallop, 2010), and workplace violence (Dellasega, 2011); however, the contextual and organizational relations between nurses and nurse managers require additional investigation. Nurses’ collective voice is rarely heard and often silenced (Canam, 2008; Pannowitz, Glass, & Davis, 2009) in an international health care context characterized by increasing demands and contracting budgets.

The purpose of our research was to explore nurses’ experiences of managing nurses and being managed by nurses. By fluidly integrating critical, feminist, and postmodern perspectives, we explored the local knowledges and clinical practices that constituted nursing management discourses within the culture of an Australian critical care unit. Our critical and feminist intent was to create opportunities for nurses to speak and consequently validate voice. The postmodern perspective contests unitary subjectivity of participants (Ogle & Glass, 2006) and valorizes alternate complex subject positions allowing alternate and subjugated knowledges and practices embedded in nurses’ experiences to be illuminated. This permits the inscription and resistance of subjectivity through discourse to be shown. As writers of this article with our own multiple subjectivities, we intentionally and freely roam between critical, feminist, and postmodern perspectives. The results of our research revealed nine subject positions adopted by participants in their working relationships. The subject position we report in this article is “junior novice.”

## Context of Nurse Attrition: The Unvoiced Discourses

The research pertaining to attrition of nurses is sparse; however, nursing management is a significant factor (Cooke, 2006; Ogle & Glass, 2006). The turnover rate of critical care nurses worldwide is between 14% and 30% (Hauck, Griffin, & Fitzpatrick, 2011; O’Brien-Pallas, Murphy, Shamian, Li, & Hayes, 2010). Nursing vacancy rates of 30% within intensive care units (ICU) are not unusual (McKenna et al., 2011). Surveys of nurses reveal low morale, poor working conditions, and that one third of nurses would leave the nursing profession if they could (Aiken, Clarke, Sloane, Lake, & Cheney, 2008).

A continual shortage of critical care nurses has resulted in strategies to attract and maintain nurses (HWA, 2012); however, complex contextual issues constitute the social reality of nursing, including oppression (Roberts, 2006), horizontal violence, bullying and conflict (Dellasega, 2011; Hutchinson, Jackson, Wilkes, & Vickers, 2008), and gender issues (Speedy, 2009). Attrition rates of new graduate nurses can be as high as 50% to 60% (VanWyngeeren, & Stuart, 2011). With nursing shortages, attention is often focused on quick solutions to recruit more nurses rather than on the historical problem of poor retention. There is a double oppression of being a female in a male-dominated world and being a nurse in a subordinated health occupation (Glass, 1998). An exploration of the culture of nursing management from the contextual clinical field that interrogates and examines discourses and practices from nurses’ perspectives is absent.

## Interrogating Discourses of Management, Leadership, and Administration

In searching organizational and nursing management texts and research, we found that management, leadership, and administration display shifting conceptions and ambiguity. There are narrow conceptualizations, with a predominant focus on management and leadership. The term *administration* is scant, disparaged, and refers only to a formal designated position or the role attached to a formal position within a hospital or institution. Leadership and management, however, refer to behaviors, attitudes, attributes, roles, and skills, which many authors have argued a nurse should aspire to and learn to excel in (Dye, 2010; Haycock-Stuart, & Kean, 2012; Sullivan & Decker, 2009). Despite the espoused notion that leadership does not require a designated formal position, with the exception of Linton and Farrell (2009), almost all of the scant nursing management research is based on “leaders” who are holding a formal management position (Haycock-Stuart & Kean, 2012). Leadership is accorded an elitist element and connected with notions of progress. The term *management* is also often denigrated and aligned with control.

Nursing texts are predominantly prescriptive of what a good nurse should do to attain management status. The normative roles and functions of planning, controlling, organizing, time management, and budgeting are antithetical to the intuitive political and communicative work of managers established in organizational research (Grove, 1997). Nurses’ experience focuses principally on senior leaders who are celebrated for their extraordinary achievements utilizing interviews or an autobiographical format to depict their lives (Smith, 2002). There is a recurring theme calling for improved leadership and leadership education (Dignam et al., 2012; Swearingen, 2009). Feminist views of leadership advocate for a shared, episodic, consensual, and relational focus, and contest the male dominant views in most leadership literature depicting the successful leader as aggressive, forceful, and competitive (Chinn, 2008). Armstrong (1992) depicted geese flying in a "V" formation with the lead bird rotating.

The notion of power is seldom linked to nursing leadership, and when mentioned has negative connotations. However, Barker’s (2002) analysis concluded that leadership, “when broadly conceptualized, is the exercise of power . . . [and] influence is the prime leadership tool” (p. 52). Power, like leadership, is a relationship between leaders and followers involving motivation, resources, and influence. Leadership has been utilized to clothe managerial positions in a charismatic mantle that reinforces and legitimates the role and social practices as natural, while detracting and mystifying unequal power relations in many organizations (Watkins, 1986). Huber (2006), within a nursing context, advocated that power and leadership be intimately intertwined because power is also the ability to exert influence over others. Burns’s (1978), in classic work on transformational and transactional leadership frequently utilized as a framework for nursing research, statedthat “naked power wielding can be neither transactional nor transforming only leadership can be” (pp. 19–20).

Girvin (1998) noted that “transformational leadership concentrates on the ability to influence situations or people by affecting their ways of thinking, even affecting their underlying values” (p. 38). Congruent within nursing, Dye (2010) asserted that “perception is more important than reality. . . . As a people orientated leader, your job is to steer others’ way of thinking” (p. 68). The notion of leadership as the ability to influence others, promotion of a vision (Jost & Rich, 2010), use of charisma, and motivating followers through communication can be aligned with the concept of the management of meaning (Fairhurst & Grant, 2010). What management and leadership really are arises from their social construction and shifting meanings attached to management to reflect social contestations (Fairhurst & Grant, 2010). Charismatic and transformational leadership have more to do with how charisma is constructed in a particular context and its cultural understanding than with any possession, property, or natural trait.

Language is used by leaders to give meaning to work, with metaphors and political language utilized to influence and motivate workers (Huber, 2006). We assert that the discourse within current leadership literature reflects a more sophisticated method of managing meaning. Motivation of staff toward organizational goals through articulated and promoted visions and values rather than coercion is dominant in the literature. This has extended to include such elements as spiritual and emotional intelligence, the creation of soulful workplaces, and transformation of a nursing culture (Dye, 2010; Jost & Rich, 2010) wherein the skilled management of emotions and worker energy are tapped.

Scholars’ writing on leadership depicts leaders as controlling the construction of reality through a monologue or "one-way street," with the assumption of superiority and control rather than a process of negotiation. Nursing literature is replete with writings on descriptive assertions that culture can be transformed with good nursing leadership (Jost & Rich, 2010) and that transformational leadership and shared governance lead to a new level of excellence (Bamford-Wade, 2010). We could not identify nursing literature with negotiated, episodic, or rotated management notions. Similar to Feldman (2001), we found an interchangeable but evolutionary shift in the terms *administration, management*, and *leadership* utilized by authors within the literature, with all terms representing similar concepts of power. This is particularly evident in the relatively uncritical stance seen in most of the nursing literature pertaining to management and leadership, and the tendency of authors of nursing literature to adopt dominant discourses and value leadership and management as superior to the value of nursing clinical practice.

Consistent with the discourse of science and rationality, the relations of managers and nonmanagers are usually assumed to be unproblematic and easily classified into bipolar categories: managers and workers. However, Alvesson and Deetz (2000) noted that the relationship between managers and nonmanagers is not clear-cut. This is because managers also work; they do things, and workers manage in the sense that they plan, decide, solve problems, coordinate, take initiatives, and exercise influence. Many nurses, including the participants of our research, are simultaneously in the position of managing other nurses as well as being workers, or being managed. Even the most senior nursing positions are accountable to another level of management. Few nursing texts, with the exception of Sullivan and Decker (2009) and Yoder-Wise (2011), acknowledge this fluidity of roles.

The competing discourses of new public management were identified by Cooke (2006) in a large study in England of nurses and nurse managers. Pronouncements of empowerment implied work intensification and tighter control as nurses voiced their distrust in their descriptions of "seagull" managers: “They fly in from a great height, make a lot of noise, drop a lot of crap, then they fly off again” (Cooke, 2006, p. 223). Discourses that predominantly inform the nursing literature include normative, positivistic, technical, functional, and patriarchal accounts, and the discourse of managerialism.

Feminist studies have investigated organizations and identified multiple layers of meaning related to nurse unit managers (Paliadelis & Cruickshank, 2008); however, additional research is required. We could not identify critical or postmodern studies of discourses, identity, or subjectivity specifically related to managing nurses or being managed by nurses in critical care. Nursing studies utilizing critical, feminist, and postmodern methodologies have elicited differing conceptions and discourses of healing and caring rather than only instrumental patriarchal discourses (Watson, 2000; Wicks, 1995). Few postmodern and feminist organizational studies of nursing have identified multiple constructions of subjectivity and explored the concept of subjectivity management (Cheek & Gibson, 1996; Nelson, 2001).

Traynor (1999) identified multiple subject positions and conflicting discourses between nurses and nurse managers within a community context. Hostility arose from nurses who resisted discourses of efficiency and utilized the discourse of caring as a moral activity. Managers reported nurses as irrational, fearful, traditional, self-interested, and unreflective. Nurse managers low in the hierarchy spoke from multiple, often contradictory positions. Subject positions identified for nurse managers were acting in the public interest, manager as therapist, revolutionary, and professional. Subject positions for nurses were not identified. Although we acknowledge the role of patriarchal and hierarchical structures of power, we did not explore the social relations within the specific hierarchy of nurse management itself. Methodologically, feminist studies have identified multiple subject positions of both participants and authors (Bloom, 1998); however, the authorship reflected a unitary self. No studies could be identified that were authored as a mobile or nonunitary self.

## Theoretical Framework and Methodology

Our ethnography was theoretically informed by critical, feminist, and postmodern perspectives. Writing from the authorial position of nonunitary and/or mobile subjectivity (Bloom, 1998; Ogle & Glass, 2006), we aimed to highlight any methodological tensions, privilege social justice, and advocate for subjugated knowledge while contesting the romance of knowledge as cure (Lather, 2000). Davies and Harré (1990) asserted the notion of position as an “expression with which to talk about the discursive production of a diversity of selves” (p. 47). We concur that “there is no one ‘true’ representation of self and identity. . . . Identities are actively negotiated and transformed in discourse . . . where strategic construction and reconstruction of self occurs” (Marshall & Wetherell, 1989, p. 125).

We intentionally moved fluidly from the desire to give voice to marginalized or silenced voices, particularly those of women (a feminist and critical perspective), to tracing and interrogating discourses, practices, and power relations that shaped nurses subjectivity (a postmodern perspective). Postmodern notions of power and discourse were predominantly informed by Foucault (1977). These discourses include “practices, behaviors, objects, technologies and concepts, all of which shape and form the body” (Threadgold, 2000, p. 50). Therefore, by utilizing this framework, our intention was to erode the fixedness of categories, to recognize the importance of blurred boundaries and multiple selves in representation.

### Critical, Feminist, Postmodern Ethnography

Critical, and to a greater extent, postmodern perspectives argue that an ethnographic text is an imperfect construct. Yet, it is the use of the lens of fluidity that enables researchers to expand possibilities of interpretation rather than closure. Researchers utilizing this approach intentionally seek partial and local truths rather than grand narratives. The perspectives from which these ethnographies originate privilege the “unsaids” (Pannowitz et al., 2009).

The embodied practice of being immersed in the field obliges the researcher to experience the contextual contingencies that play on participants. From a critical perspective, research is driven by an emancipatory principle that recognizes material and cultural practices that create oppression. These, in turn, create space for multiple voices to speak and/or be represented (Denzin, 1997). If the researchers edit themselves into the text complete with their presuppositions and biases, if they enter into dialogue with the participants in an attempt at mutual understanding and gaining multiple insights, then ethnography can become a vehicle to promote change and a journey of silenced possibilities (Sultana, 1992).

From a feminist perspective, reflexivity and emotionally safe and trusting relationships between the researcher and participants were of utmost significance. Within our research, we attempted to address power imbalances by spending time with nurse participants to gain an understanding of each as a person. Issues that arose in the clinical context were clarified and reflections about the environment shared. Participants were encouraged to select the site for their interview and to edit their transcript. Consistent with the openly ideological feminist goal, the feminist conception we brought to this ethnography was to correct both the invisibility and distortion of female experience (Lather, 2000). The postmodern ethnographic perspective focused on rethinking identity and culture as constructed and relational. Therefore, the three perspectives combined to privilege the particular subjectivities of nurses and the discourses that have previously rendered aspects of their work invisible.

### The Ethnographic Method

Conducted over a period of 10 months, we sought direct participant observation, individual interviews, and reflective field notes to comprise the data. In addition to the authors, 11 registered nurses from all levels of the nursing hierarchy participated in the study. One author extensively entered the lived world of the participants and their culture by observing, talking, recording, and “being with” the nurses. Rather than denoting culture as static, we viewed culture as fluid and as a social construction with contested communicative processes, and the discursive production of competing discourses was seen as written, spoken, or imaged texts (Threadgold, 2000). Postmodern notions of culture centered on the changing, fragmented, and spontaneous interpretations by organizational members rather than a deep unified truth embedded in a seemingly linear and progressive history. Utilizing critical, feminist, and postmodern ethnographic approaches, we focused on the importance of embedded intertextuality to reveal nuances of discipline and governance (O’Byrne, 2011).

Participant observation and in-depth interviews were utilized to generate reflective field notes. Local knowledges and clinical practices that constituted multiple discourses and culture and inscribed participant subjectivity and bodily experience were explored and interrogated. Data analysis included examination of discourses to elicit how truth was defined and by whom, and the assumptions, effects, contradictions, silences, and social practices necessary for the existence of the discourses. Practices surrounding the discourses were mapped, including power relations and the reinscription of discourses by clinical practices. Simultaneously, marginal and subjugated discourses were identified and highlighted in the form of alternate or oppositional knowledges and practices. Rather than attention to genealogical aspects, our discourse analysis, informed by Parker (1992), was focused on how participants were inscribed by or resisted various discourses, and which subject positions were adopted.

Data were analyzed inclusive of the authors’ fluid subjectivity and freely moved between critical, feminist, and postmodern perspectives. Our critical exploration aimed to subvert the apparent naturalness of relations in management and to raise possibilities for alternate conceptions. In our analysis, feminist insights critiqued the inscription of gender and interrogated the constructions of female identity, and therefore made space for nurses to speak. The postmodern perspective subverted the notion of the unitary subject that is rational and transparent. We sought to displace the coherency and presumed free development of social relations so that the reproductive processes thatstructure the self, prohibit self-differentiation, and create a false consensus with others could be identified.

### Site Selection and Access

The site was selected on the basis of a telephone call from a nurse requesting that she and staff from the unit she worked in be involved in a research study. This correlated well with one author’s clinical and academic background in critical care nursing and health administration. Both the hospital and university ethics committees granted approval. It was required that the researcher be sponsored by a hospital senior staff member. One author telephoned, emailed, and met with numerous senior nursing staff members, outlinedourintentions for the study, and was readily given support. Maintaining continued access, including the extent of access, was important, particularly with respect to being informed about different meetings and their perceived significance.

### The Participants

Of the 11 nurse participants, most read the plain language statement that was placed in the communication book or attended one of several informal talks. The specific criteria for participation were that participants were registered nurses with the (then) Victorian Nurses Board and that their employment was directly related to the critical care unit in a clinical or managerial position, or both. Seven clinical nurses were bedside nurse clinicians or clinical nurse specialists; the other 4 participants were from the nursing hierarchy that encompassed associate unit managers, unit managers, nursing supervisors, clinical directors, and directors of nursing. The exact positions of each of the senior nurses are not specified to safeguard participant confidentiality. Of the 11 participants, 9 were women and 2 were men.

### The Locale of the Intensive Care Unit

We undertook our research in a Level-III general ICU (College of Intensive Care Medicine, 2010) that comprised one ward of a large public teaching hospital in Melbourne, Australia. The unit cared for adult patients with complex critical illnesses including general medical, surgical, trauma, neurological, and road trauma. The unit physically contained between 10 and 16 bed spaces; however, available or “open” beds depended on patient requirements, funding, and nurse availability. Between 80 and 100 nurses were rostered within the unit, most of whom were required to work rotating shifts. The nursing staff employed within the unit comprised one unit manager, several associate unit managers, clinical nurse specialists, a nurse educator, and clinical nurses who were all registered nurses, some undertaking critical care studies.

## Data and Discussion: Nurses’ Multiple Compositions

We organized the data comprising participant observation, interviews, and reflective field notes into nine identified subject positions adopted by nurses. The subject positions were junior novice, detached unemotive individual, pleaser, exceptional and elite, expert clinician, emotional human, personal improver/coach, keeper of order and appearance, and strategist. In this article, we report on the dominant subject position, titled junior novice. Subjugated discourses are included within the subject position to display the multiplicity of perspectives, the practices, and discourses that informed and reinscribed the subject position and the effects and fluidity of individual subjectivity. The subject positions are our interpretation and do not negate that additional interpretations are possible and likely. We did not attempt to explain away situations or to create fixed meanings, such that contradictions are frequently exposed and left in tension to highlight the complexity and keep open the notion of multiple possible realities.

### Shifting and Fluid Subject Positions

We found subjectivity to be partial, fluid, and nonunitary as participants freely moved between subject positions, frequently adopting numerous positions. Individuals were not constrained to be informed by every subject position and frequently adopted conflicting positions. In their interviews, participants sometimes expressed concern that they held conflicting opinions. Other conflicts were evident following analysis of the data. Data pertaining to one subject position also frequently displayed an overlap with other subject positions, thereby producing the webs of connection that supported and reinforced the preeminence and seeming naturalness of dominant views and discourses. Because the first author of this article was a participant as well as a researcher, her reflective field notes are included. Therefore, there are examples of her situating herself into subject positions as part of the data.

General observations of the unit and the fluid movement of subject positions were noted from the researcher’s first shifts in the unit. Some uncertainty with her positionality was also apparent:

The unit is busy, noisy, and cluttered. Beds are lined up in rows, each containing a body tenuously attached to a vast assortment of machinery. [A participant] told me about the lady who she was caring for who had suffered major trauma, including a serious degloving injury. “She incurred a head injury getting out of a stationary taxi.” Her tone was both cynical and mocking. . . . I know this is typical ICU talk and it does not mean she does not care, but from a researcher position it seems very stark. She proceeded to the head of the bed and gently wiped the lady’s face. Leaning her own face close to the lady’s ear she slowly explained to the lady who she was and that she would be looking after her. . . . So why did she adopt such a cynical position when she talked with me?

### The Junior Novice

We describe this position as an inferior or subordinate position whereby the nurse adopted a position of being less knowledgeable, having limited experience, of being inferior to, and of being dependent on others. Junior novice was frequently adopted despite participants’ extraordinary nursing and medical knowledge and their complex clinical expertise. This was not a position adopted by junior nurses; rather, it was a role of being personally inferior to another.

Junior novice might reflect that the participants were predominantly female and therefore had been previously socialized into positions of subordination. Oppression and subordination of both women and nurses has been well documented (Almost, 2006; Dellasega, 2011; Roberts, 2006). Many participants were aware that they adopted this position and talked about specific issues for themselves, such as lack of confidence and the problem of gender in nursing; however, the researcher noted the extent of this subservience as being remarkable:

I thought all critical care nurses were a bit more assertive. It is a shock to find that these nurses lack so much confidence. Today I walked into a room to find two nurses reading the charts in a corner of the room while the medical staff discussed the patient at the end of the bed. . . . When they left the nurses complained to each other that no notes were written and they had no idea of the plan of care. When I explained my astonishment to them that they were excluded, they shrugged it off as being undesirable but quite normal.

Another participant articulated her position of inferiority when she described her experience of her performance interview with a male nurse manager:

I did feel intimidated by him but . . . it probably wasn’t entirely his fault, but a lot of it is my background and upbringing. Sort of being very respectful of authority and . . . in a way putting authority up on a bit of a pedestal. . . . So I find it just difficult to be, I guess normal, and talk about everyday things with him.

The conversation reflected and was informed by the managerialist and patriarchal discourse that privileges management as a highly complex superior occupation, compared with her own activities. The nurse identified herself as not being able to be normal, and therefore she owned the responsibility for the perceived self-deficit. The practice of educating critical care nurses through a postgraduate critical care course reinscribed this position. When directly asked if thecourse increased her confidence, she replied as follows:

No, not really . . . because you get totally analyzed and I don’t think [this hospital] is the most encouraging place to be at for your course, although I think it is a good place to do it. I think you come out being very competent . . . [but] my problem is sometimes I have a hard time believing it.

This was consistent with the literature regarding the reinforcement of inferiority and subservience within clinical environments and hospital training programs for nurses (Nelson, 2001). It has also been documented to occur in university programs. In relation to undergraduate programs, Jackson et al. (2011) found that although it was expected that nurses would be empowered, in practice this did not occur. The effects of subordination were also infiltrating nurses undertaking postgraduate education.

A postgraduate nurse participant asked the researcher to attend her appraisal meeting with the nurse manager. She reflected,

Her nervousness was so obvious. She sat in the manager’s office, her back to the door, facing the manager directly. Her feet swung in circles under the chair and she fidgeted with her hands, at times sitting on them to almost stop them moving. She really had difficulty in getting out the words she wanted to say. However, having something written down seemed to help and she was determined, almost self-righteously, to say something. The manager, however, was quite cool, sat in his chair with his feet stretched out, and I think in his way [he] tried to support her to say what she wanted. When he turned around, however, and switched on the computer, bringing up documentation of her past appraisals and reports, I wondered what effect her verbal feedback really had when what she said was not in any way documented.

The nurse discussed the importance of having the researcher present for the appraisal:

I knew you were there to observe relations between management and grass-roots nurses, so I thought you might appreciate the opportunity of just seeing a meeting where we discuss some of those issues. . . . Maybe it did boost my confidence a little bit . . . just having someone else there. Yes, it could validate what I was saying.

The textualization of knowledge, including who had access, who performed the documentation, and what was documented, became a mechanism for creating or constructing what might be taken as reality, but that also reinforced inherent power relations. The nurse’s comments to the manager were never documented and appeared as a token, which enabled managers to state that staff were encouraged to give feedback on managers and to raise issues. Another nurse participant spoke about her appraisal:

I was really scared about [my appraisal], like when I went to [my manager] at my instigation to get it done. . . . He sort of said, “How do you feel about it?” . . . And I said, “I am really scared about it, you know.” He said, “Why is that?” I said, “Oh, I don’t know. I think that most things that will come up I’ll probably already know about.” But . . . you always worry there’s something that will come up that you . . . don’t know about.

The practice of appraisals appeared to be a game whereby participants adopted certain positions. Managers and clinicians knew there was a power differential; however, the game was played without this being articulated. Denying clinicians’ awareness of the power imbalance, however, reinforced nurses’ subservient position, inclusive of the act of self-instigation of the appraisal. That they were scared became another self-deficit. Participants fulfilled and enacted these roles, reinforcing their own subject position in a self-referring cycle.

The experience of staff appraisals has previously been documented to be one of mistrust by nurses, who reported concern that this could be used as a weapon by managers (Redshaw, 2008). Nurse managers also acknowledged expectations to fulfill their own position of superiority. A peer-review process created a sense that colleagues were also involved in the appraisal process; this softened the other power relations inherent in the assessment. The peer-review process considered how the assessment took place, if it was verbal or documented, where it took place, and also the relative position of individuals in the discussion. The requirement to tentatively balance power relations so that it was not absolute but sat in tension aligns with Foucault’s (1977) notion of power. The peer-appraisal process allowed for colleague input; however, it was also a mechanism that encouraged internal policing of nurses’ behaviors and enhanced individual self-surveillance.

New nurses were often unaware of the behavior expected, particularly those who had not completed a postgraduate course. A meeting occurred between a nurse manager, an educator, a nurse who was new to the unit, and her preceptor to evaluate the orientation of the nurse. It was evident that the new nurse was expected to be subservient. The researcher reflected,

This nurse was really upset. In the meeting, when asked how she thought she was performing, she replied that she thought she was doing okay. The educator then asked her how she had formed this opinion. The nurse replied that the relatives had been happy with what she had done and had thanked her many times. The educator then informed her that . . . she did not seek sufficient assistance or communicate to others what she was doing. Tears welled in the nurses’ eyes and she did her best to continue the conversation, which was informing her that she was not performing to expectations.

The negative attitudes within the clinical environment were so pervasive that the researcher realized she also slipped very easily into a subservient position at the appraisal meeting:

I sat there dumbfounded and shocked at the brutality of the meeting [with] the educator, whom I had expected to be a supportive person. . . . The situation [was] a type of public humiliation. . . . Why didn’t I speak up at the time? I did agonize, but felt immobilized that I would be told I did not know the details and my role as researcher would be questioned. My silence prevailed. which gave my apparent assent.

During her interview, a clinical nurse described one of her first shifts in the unit. She commenced employment in the unit having completed a critical care course elsewhere:

I was there very slowly and taking my time. . . . [I] had to change the hemofilter and probably only once had I ever used one. And the nurse in charge bowled in and said, “That hemofilter was supposed to go on. It was quite clear at the eleven o’clock round.” . . . I just felt like bursting into tears . . . This was what her attitude was always like.

The subordinate position was reinscribed by unit practices that valued efficiency and precision, with little regard to the individual. Routine surveillance by more senior staff identified defects in ward routine. There was an assumption that the nurse must be able to circumvent any possible deviation to the routine, and this assisted the reinscription of the subject position. Nurses felt ineffective and inadequate.

The novice or subservient position was not specific to clinical nurses, but also to nurse managers. After attending a hospital executive meeting to observe senior nurse managers, the researcher made the following note:

The 20 or so seats are arranged in a big circle around the room with tables in the front of the seats. Each nursing co-director of a directorate is seated alongside their respective medical co-director as in partnership. My understanding is that these senior nurses are very proud that nurses fill all these co-director positions. Reports for each directorate are given by the respective medical co-director, with the exception of one directorate where it is given by the nurse. The doctor has already left.

The inferior position was evident in a nurse manager’s interview. She elaborated on her feelings of uncertainty with her position:

Others have worked with the executive a lot more. . . . Some of my limitations are understanding the really big-picture stuff. You know, looking at developing what services we are going to need in our whole directorate. Being able to be lateral and have a helicopter view. . . . That’s their expertise. They have that helicopter view, and they are all coming up with policies and, you know, different strategies. Strategic planning, I don’t think it’s out of reach, but it is something I am going to have to develop.

The managerialist discourse of policy development and strategic planning prevailed to support management as a superior, highly complex, and highly knowledgeable occupation compared with what she perceived her current skills to be. Her views on management were evident also in her description of the development of a new nurse manager. Her description equated with novice and displayed her view that the practice of being tough as a manager was a positive sign of growth and confidence. When she commenced as a manager, “I was a little bit tentative. . . . It’s really difficult. Do I be friends with these people or don’t I? When do I take the tough stand?”

This was supported by the patriarchal discourse that equated management with positive masculine traits of decisiveness, toughness, and lack of emotion. The nurse manager commented that she was aware of gender issues and talked about her concern of being treated like a little girl by men. Other participants grappled with issues of gender. A clinical nurse commented on how she viewed nursing as a process of selecting predominantly “girls” and how this contributed to the subordinate position of women in nursing and expectations of nurses’ behavior:

It’s a bit of self-selection . . . old-fashioned girlie issues that were put with feminine people. . . . I don’t know why I [lasted]; tenacity it must be. . . . If you say to any female nurse, “They’re a real boy nurse,” we all know what we mean. . . . They’re messy, sloppy, and they try to get away with charm. They rise up the ranks because of charm, not because of hard work or any great aptitude. . . . Women are harder on women. They expect different behaviors of men and of women.

The nurse’s talk centered on the compliant “girlie” behaviors expected of females, their subordinate position, and the institutional privileging of males. Males in nursing might be socialized like females because of the gendered nature of the occupation. Her explanation of the institutional subordination of women as being a problem of women who become bosses, however, also perpetuated the patriarchal notion that judges the behavior of women more harshly than men (Brescoll & Uhlmann, 2008). Her clinical experience refuted this notion.

The subordinate position was frequently assumed by most nurses and disciplinary actions were invoked on those who appeared to step outside this position. During the time of the study, 10 of the 11 nurse participants were required to reapply for their own or another position. It was generally assumed that most would regain a position; however, it was known that one management position was being shed and nurses were being moved to wards. The process resulted from a hospital restructure and served as a mechanism or technique for control.

The following reflection was given by a male nurse manager regarding an incident that occurred shortly after he was appointed as a nurse manager. The reflection indicates how he learned to change his behavior, and his dilemma of whether this change was good:

[I] did something on my own without informing various managers. . . . I was so passionate about this . . . and I was getting enormous pressure from my staff. . . . I was certainly relaying that onto my management group and above . . . and sort of hitting a brick wall. My perception was that they weren’t listening. . . . I got my wrists slapped for it in no uncertain terms. . . . Two years down the track would I do the same thing? . . . I maybe have become more comfortable that you can’t just do everything. . . . I’ve got to sort of be in line with hospital processes as well, and be that meat in the sandwich.

Nurse managers experiencing a lack of organizational support has been documented in the literature (Regan & Rodrigurez, 2011). This has not, however, included lack of support and poor interpersonal relations with other nurse managers. Feelings of inadequacy, accountability without authority, and stress because of being torn between staff and upper management, without the support of either group, has been documented (Cooke, 2006). The quandary of nurse managers to change their behavior to be more subservient and act “dumb” has not been documented.

The subject position was also reinforced by the notion that nurses were not intellectually bright or intelligent individuals. Within a managerialist discourse, managers are aligned with a superior and elite status, and higher intelligence is described as a common trait among leaders (Bligh, Kohles, & Pillai, 2011). For nurses, despite many havinga university education, their image is one of not being knowledgeable or scholarly (Milisen, 2006). Frequently in our research, nurses referred to other nurses as being idiots. This opinionwas evident in the discourse of a nurse manager:

We are not getting the best and the brightest anymore because of the sociological changes, the options for women. . . . To be honest, I wouldn’t have been a nurse if I had been born twenty or thirty years later. . . . I know how I ranked in my class. . . . We did get the best and the brightest. We might have pounded it out of them because they had to conform to the hierarchy, but I think they were still very intelligent people.

### Marginalized and Subjugated Discourses

Two marginalized and subjugated discourses also supported and informed the subject position of junior novice. Subjugated discourses were most evident in participants’ individual interviews when they were reflecting on issues. One marginalized discourse was of equality and team relationships, and valuing these over superior–inferior or hierarchical relationships. Skills of management were not privileged over those of a clinician, but instead were viewed as different skills. Viewing management as superfluous and unnecessary was a second subjugated discourse.

#### Equality and team relationships

A clinical nurse explained her view that a manager needs to be one of the team, like all other nurses, and he or shecan belong to or be owned by the team rather than owning the team:

People will work better as a team if they see you as part of their team. When you become a manager you are not really part of anybody’s team anymore, and you don’t belong to the girls on the floor. You don’t belong to the nursing management hierarchy because you are just the plebe . . . managing the nurses.

A nurse manager talked about resistance from nurses to other nurses who take up management positions, and her view of management as not being better than clinical nursing but being a role for nurses:

Thinking about nurses as being in a position to run hospitals is pretty remarkable. A nurse executive officer said that the most difficulty and opposition [she experienced] had been from her own nursing board. I guess to me it’s not that you’re going beyond nursing or you’re better than . . . it’s that you’re using your knowledge and skills, which are very closely tied to the core business of the hospital.

One nurse manager, who described occasions when she interacted and joked with other nurses as equals, supported the importance of a team; however, she still referred to ownership of meetings. She also rebuked some male leadership experiences:

Sometimes in my meetings you wouldn’t know who was [more senior] sitting around the table. None of that matters to me. . . . You need groups that have people with different sorts of roles . . . and as long as my boss tolerates that and I don’t get any messages that I need to be more like him . . . we are quite a good balance.

Marginal discourses were also sometimes interjected among managerialist discourses. Another nurse manager predominantly articulated discourses that supported hierarchical relations; however, in her interview, she articulated a view of how she acquired her managerial skills,whichshe would have been unlikely to document formally. Dominant discourses of management are that it is a highly skilled activity taught and learned from senior management mentors or via university qualifications:

My mother, in particular, was very much about developing relationships with all sorts of people. . . . She had a respect for all those people, and there is no doubt in my mind that I picked it up from her.

The marginal discourse of management acquisition would have been slightly more acceptable to dominant views on management had “mother” been replaced by “father.” Not all nurses willingly adopted the subordinate position, and expressed concern that relations with nurse managers were worse than poor relations with doctors. A clinical nurse explained her experience on a committee external to the hospital:

I was certainly the only full-time clinical person [on this committee]. Our [nurse manager] was on it, and the power plays were just incredible. She wouldn’t speak to me. . . . She might sort of nod to acknowledge me, but there was no conversation . . . and yet the thinking is that doctors are oppressing nurses. . . . It was the nurses that were very down-putting and rude, basically.

Another clinical person also described the importance of teamwork, and of staff supporting each other. The discourse of economic efficiency with large-sized units dominated over subjugated concepts of staff support and interpersonal relations:

Above our nurse manager [they] are pushing for more and more beds. . . . Everybody was saying we’re going to be saving more lives, [but] . . . you can only push people so far. . . . You’ve got people doing extra shifts, people doing double, agency people being contracted. . . . You need to have support and we don’t have it, but we keep doing it. . . . You do get a very high turnover of staff. . . . You can come on to a shift where you are in charge . . . and you can have four to five people that have been in the unit for a handful of months, and . . . you’re expected to support [them].

#### Management as superfluous and unnecessary

The discourse concerned just tolerating management and viewing it contemptuously. It was not perceived as superior to clinical nursing. This was usually expressed only by nurses who exhibited some personal self-confidence. Concepts of management were frequently viewed in this discourse as rhetoric, and holding a management role was not assumed to indicate either greater personal ability or prestige. One clinical nurse reflected on her experiences with a nurse manager, displaying some skepticism and contempt:

I think he was threatened by me. . . . I always had to approach him for things. . . . He interviewed me . . . when I first went there. I think I must have preempted his three questions because . . . he said, “Well, I have got nothing else to ask you,” and sort of looked at me. . . . I think he was immature for the position.

The same nurse described her views of management with respect to being offered a new position at another hospital. She was confident of her ability to fulfill the new position offered; however, she questioned the assumption that she should want the new position:

[They] have given me the clinical nurse specialist description and want me to apply. . . . “Well,” I said, “you have got to sell it to me harder.” . . . I am challenging what it’s about. Well yes, I am worthy of it, but does it fit well with me? Is it really something I want to commit to?

The nurse questioned the desire to adopt what was assumed to bea prestigious identity. Her challenge of this dominant view was met with some incredibility. Her potential move from what she perceived as more prestigious to the offered position might have raised her awareness of the conflicting processes involved in forming her identity. She also indicated an awareness of “game playing.” The nurse, however, viewed game playing as separate from the real world, and she indicated a strong sense of individual freedom in the game rather than any notion that the real world could similarly be a construction. The legitimacy of the articulated discourses of nursing management was also challenged by another nurse, who believed that much of what was articulated was accepted because of a lack of understanding by other nurses:

I think [the nurse manager] was employed to change things. . . . She is very good at rhetoric, and she won them on rhetoric. Unless you really know what she is talking about, you wouldn’t know that it’s a whole lot of crap.

The acceptance of and lack of challenge to what was articulated were consistent with the subservient junior novice subject position. There was little challenge to what was articulated. The reality was poorly contested, and was instead constructed from dominant discourses that supported existing power relations. Although the articulation of the nurse can be viewed as unconventional or conventionally unscholarly, it reflects the dispute of discourses quiteclearly.

A nurse manager indicated that he was aware that not all nurses on the unit valued nursing management, and that many were skeptical of the management role:

I don’t think they fully appreciate what is involved [in management]. . . . whether that’s a failing on our behalf because we don’t actually sell what our role is or that we don’t communicate it. . . . I don’t think they expect me to be that clinically focused, which has always staggered me. . . . Someone recently said to me when I spent a number of days out in the ward . . . “You have been out of your office a lot lately. Haven’t you got much to do?” . . . One of my associate charge nurses, she said, “How about you go and do your job and let me do mine,” which was again, that I shouldn’t have much involvement in the clinical management of the ward.

The nurse manager indicated some rejection by clinical nurses of his ability to practice clinically. The individual manager was rejected in person, delineating distinct boundaries of expertise. This was a mechanism of resistance to management processes. Given time constraints and the dominant discourse that privileged management functions over nursing practice, maintaining clinical expertise was difficult for managers.

The ability to challenge management thinking was hampered for nurses by lack of knowledge of the agenda, and by a lack of confidence in their articulation skills. One clinical nurse spoke about his experience of nurse managers:

[Nurse supervisors] come around and collect all this information. . . . They are really not understanding. . . . They’ve got a role in coordinating things in the hospital, but you don’t really see anything tangible. . . . I don’t think that they are really nurses. I don’t get a sense that they are leading. . . . We don’t see them or hear anything real. [The nurse manager] has had meetings in the unit, and some are really boring meetings, but in terms of actions and outcomes it’s not probably a productive meeting. . . . I’d say she’s not directly supportive of me personally or the area that I’m working in. . . . To be a leader and change things, surely they need to have support and understand the people. . . . If people are not satisfied with the changes then they often voice their displeasure by leaving.

Researchers have attributed poor self-esteem and confidence in nurses as a significant factor in women not gaining more senior positions within health care (American College of Healthcare Executives, 1996). Our data support the notion of nurses assuming subordinate positions. The subject position was actively reinforced and reinscribed by numerous unit and organizational practices, including peer appraisals, meetings, routine surveillance for defects in ward routine by self and senior staff, and the processes involved in undertaking a postgraduate critical care course. Disciplinary actions, including lessons in process and the need to reapply for existing positions, overtly enforced this position. Subtle concepts such as aligning nurses with poor intelligence also reinscribed this position.

## Conclusion

We set out in our research to give voice to nurses and investigate nurses’ experiences. We found that nurses and nurse managers experienced feeling abnormal, angry, and rejected. Interprofessional relations reflected a lack of individual valuing. The subject position of junior novice was primarily informed by dominant instrumental, patriarchal, and managerialist discourses that homogenized the identity of nurses and defined the meaning of “normal.” Management activities were deemed superior to the activities of a clinical nurse. Marginalized and subjugated discourses included notions of teamwork rather than hierarchical relations, equality, and a contemptuous view of management as a superfluous rather than a superior occupation. Patriarchal behaviors were contested. Previous researchers have highlighted the ideal nurse as submissive, implicitly unquestioning, and with internalized self-discipline (Nelson, 2001; Reverby, 1987). Cheek and Gibson (1996) identified the docile nurse and the discursive construction of that identity.

Our data confirm that little has changed in the social construction of nurses within the clinical environment, except perhaps the technologies that enforce and reinscribe this subject position. Previous studies identified that subordination to the practice of medicine, socialized suppression, and the perpetuated myths regarding nursing eventually undermine nurses’ self-image and confidence in themselves (Siebens et al., 2006). In our research, we found that the relations between nurses and nurse managers undermine nurses’ self-esteem and confidence. Although some resistance was apparent, nurses informed by dominant organizational discourses actively participated in constructing and reinscribing their own subjectivity and submissive identity.

The major implications from our research for nursing as a profession are increased awareness for nurses to explicitly value their own practice. Nurses need to foster a culture that genuinely permits individual diversity to alter the existing pre-scripted relations that constrain theirability to engage in more meaningful interpersonal relations. Questioning current discourses and practices that value specific economic and scientific knowledges, support patriarchal behaviors, and silence nurses is essential. The articulation of alternative discourses that value women and nursing is crucial for reconstructing a reality that does not result in women and nurses feeling abnormal, rejected, and alienated. This is particularly significant within the context of a nursing shortage.
